# Effects of rodent abundance on ticks and *Borrelia*: results from an experimental and observational study in an island system

**DOI:** 10.1186/s13071-024-06130-x

**Published:** 2024-03-27

**Authors:** Nosheen Kiran, Ilze Brila, Tapio Mappes, Saana Sipari, Yingying Wang, Erin Welsh, Eva R. Kallio

**Affiliations:** 1 Department of Biological and Environmental Sciences, University of Jyväskylä, Jyväskylä, Finland; 2 Ecology and Genetics Research Unit, University of Oulu, 90014 Oulu, Finland

**Keywords:** *Ixodes ricinus*, *Borrelia burgdorferi*, *Borrelia afzelii*, Nymphs, Infection prevalence, Host abundance

## Abstract

**Background:**

Lyme borreliosis is the most common tick-borne disease in Europe and is often caused by *Borrelia afzelii,* which is transmitted by *Ixodes ricinus* ticks. The prevalence and abundance of infected ticks fluctuate in time and space, influencing human infection risk. Rodents are reservoir hosts for *B. afzelii* and important feeding hosts for larval ticks. In the study reported here, we examined how variation in rodent abundance is associated with *B. afzelii* infection prevalence in ticks, the density of nymphs (DON) and the density of infected nymphs (DIN) in the following year. We further analysed the relationships between the abundance of infected rodents and nymphal infection prevalence (NIP) and DIN.

**Methods:**

We conducted a study that combined experimental and observational approaches on 15 islands (10 small islands and 5 large islands) in Finland. On all of the islands, ticks and rodents were monitored and sampled during the summer of 2019, with the monitoring of tick abundance and sampling continuing into the spring of 2020. On five of the 10 small islands, captured rodents were removed from the island (“removal” islands), and on the other five small islands, captured rodents were released back to the trapping site after marking and sampling (“control” islands). On the five large islands, captured rodents were released back to the trapping site after marking and sampling. The presence of *B. afzelii* from nymph and rodent samples was examined.

**Results:**

The results of the experimental study showed that neither treatment (removal), rodent abundance index nor abundance index of infected rodents in 2019 was associated with DON, NIP or DIN in 2020. Based on data from the observational study, the NIP in 2020 decreased with increasing rodent abundance index and abundance index of infected rodents in 2019. However, the DIN in 2020 was not associated with the rodent abundance index or the abundance index of infected rodents in 2019. In addition, in the observational study, DON in 2020 increased with increasing rodent abundance index.

**Conclusions:**

Our results suggest that low rodent abundance during the tick activity period is not sufficient for reducing the disease hazard and, hence, rodent removal may not be a feasible control measure in natural ecosystems.

**Graphical abstract:**

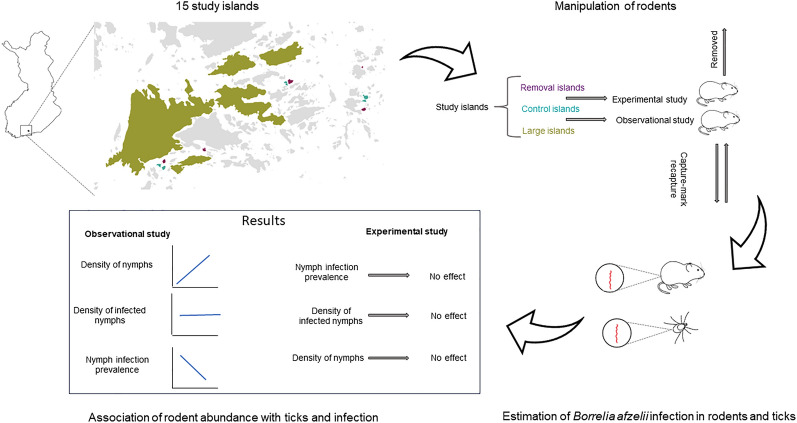

**Supplementary Information:**

The online version contains supplementary material available at 10.1186/s13071-024-06130-x.

## Background

Tick-borne diseases are a source of serious health concerns in Europe [1–3]. The most common tick-borne disease in the Northern Hemisphere is Lyme borreliosis (LB) [4, 5]. LB is caused by spirochete bacteria belonging to the *Borrelia burgdorferi* sensu lato (*B. burgdorferi* s.l.) complex that are transmitted among vertebrate hosts by ixodid ticks [6]. *Ixodes ricinus* is the main vector of *B. burgdorferi* s.l. in Europe [7]. The life stages of this tick differ in terms of host preferences, with rodents being common hosts for larvae, nymphs parasitizing a wider range of hosts and adults preferring large hosts, such as deer [8–10]. The nymphal stage is considered to be the life stage posing the highest risk to humans due to its small size of nymphs, their potentially high abundance and the potential for nymphs to carry pathogens [11]. Therefore, the local abundance of infected nymphs is considered to be a primary environmental risk for humans [12, 13].

Rodents not only provide a blood-meal source for immature *I. ricinus* ticks but also contribute to the propagation of horizontally transmitted tick-borne pathogens; uninfected larval ticks may acquire pathogens from infected reservoir rodents that acquire the infection from infected nymphs [14–16]. In Europe, one of the most common etiological agents of Lyme borreliosis is *Borrelia afzelii* (a genospecies of *B. burgdorferi* s.l.), which is maintained by rodent hosts such as mice and voles [15, 17–24]. The role of rodent abundance in the prevalence of *B. burgdorferi* s.l. infection in nymphs has been examined in several studies carried out in North America [25–27] and Central Europe [28, 29], with the results suggesting that a higher abundance of reservoir rodents translates into a higher infection prevalence in nymphs the following year. However, the Lyme disease system in North America and Central Europe differs from the system in Northern Fennoscandia in many ways. For example, occasional masting increases rodent densities in temperate Europe, whereas in northern Fennoscandia, vole-dominated rodent populations show seasonal and multiannual density fluctuations characterized by very low densities over extended periods (over the breeding season) [30, 31]. It remains to be quantified whether these very low rodent densities affect the dynamics of rodent-associated tick-borne pathogens and infection prevalence in ticks, as is expected because the rodent density fluctuations have been shown to translate into human Lyme disease incidence with time delays in northern Europe [32, 33].

Most studies addressing the role of rodent density in determining the prevalence of *B. burgdorferi* s.l. in ticks are observational or theoretical in design [26, 27, 33, 34]. Only recently has an experimental study, conducted in small outdoor enclosures in temperate Europe [29], shown that an increase in rodent density increases the prevalence of rodent-associated tick-borne pathogens in ticks the following year. Our study aimed to quantify whether rodent density variations, especially low rodent abundance, are associated with changes in *B. afzelii* infection prevalence in ticks the following year. In our study, we utilized a natural island system and incorporated both experimental and observational study designs. We investigated the associations between rodent abundance and the abundance of infected rodents in year one (t) and the density of nymphs (DON), the prevalence of *B. afzelii* in nymphs (referred to as nymph infection prevalence [NIP]) and the density of infected nymphs (DIN) in the subsequent year (t+1). We hypothesized that: (i) rodent removal decreases NIP the following year (NIP_t+1_) and (ii) the higher the rodent abundance index or abundance index of infected rodents, the higher NIP_t+1_. We also predicted that: (iii) a higher rodent abundance index will also result in a higher density of nymphs in the following year (DON_t+1_) because rodents are considered to be important feeding hosts of larvae. Consequently, we expect that: (iv) the rodent abundance index would increase the DIN the following year (DIN_t+1_), which in turn determines the level of *B. afzelii* hazard to humans.

## Methods

### Study area and design

**Fig. 1:**
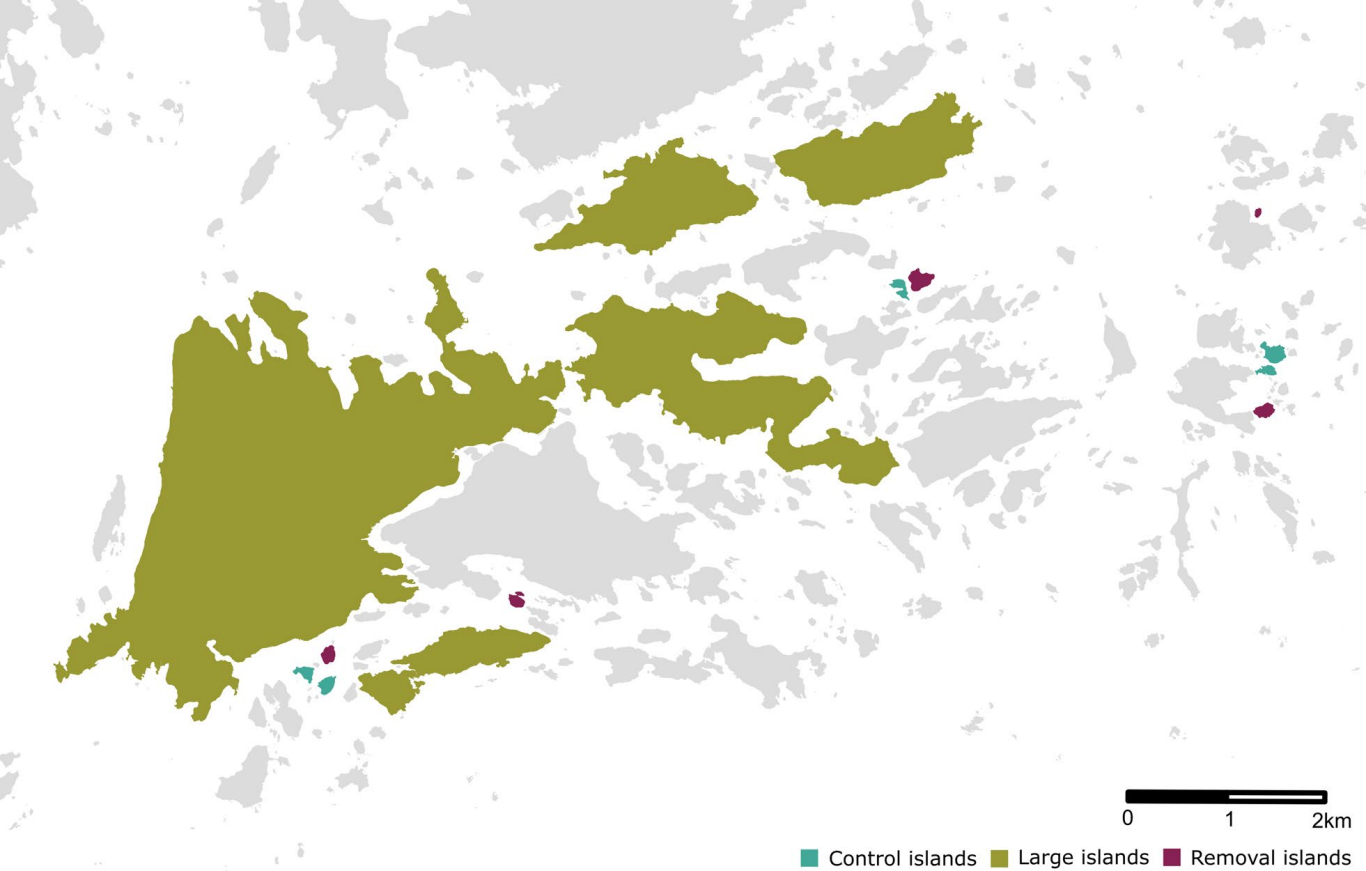
Map of the study sites showing rodent removal, control and large islands. Grey islands have not been included in the study

**Fig. 2:**
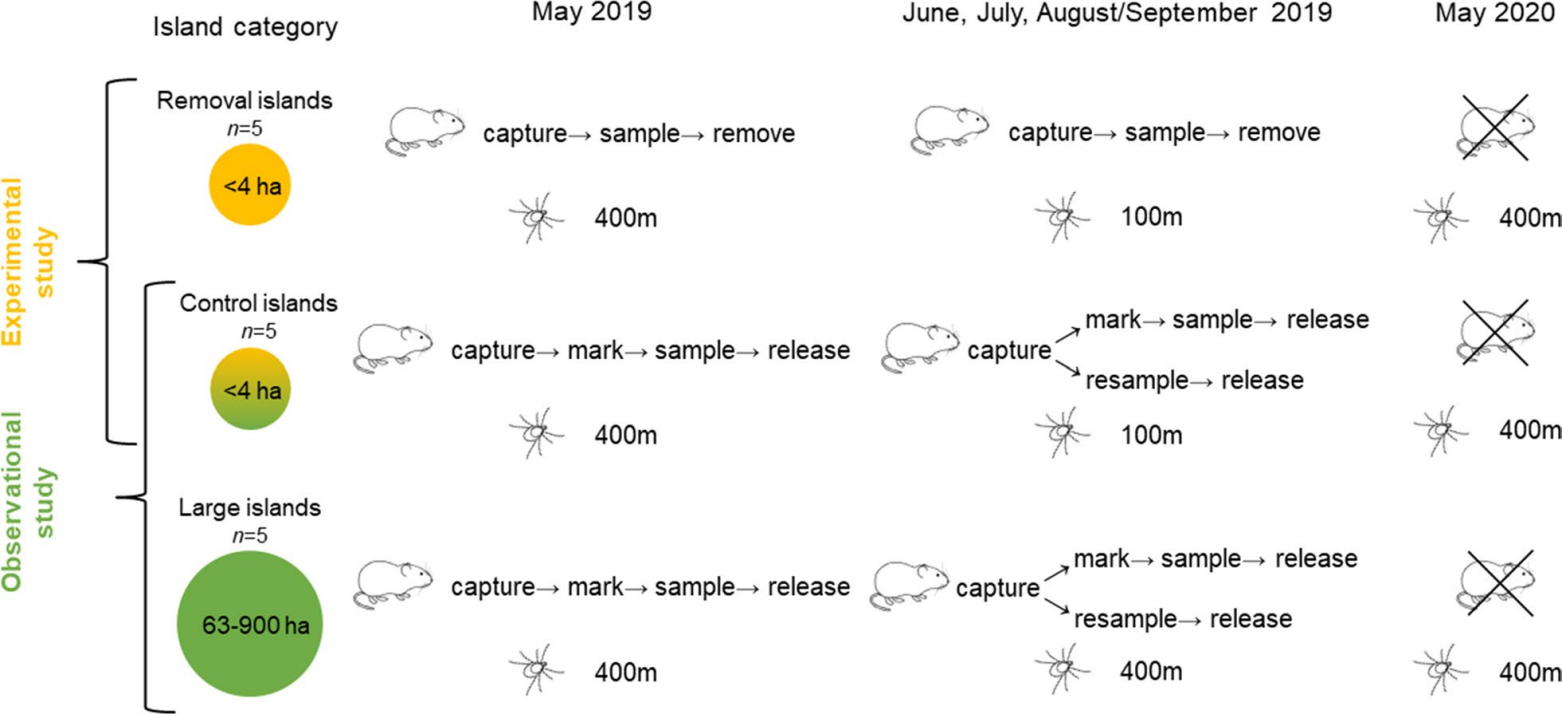
Study design including a collection of ticks and rodents in the experimental (yellow) and observational (green) studies to examine the associations between rodents, ticks and *Borrelia afzelii*. The crossover indicates that no rodent trapping was done in May 2020. The distance in meters indicates the tick collection distance on the islands

The study was conducted on 15 islands in the Porvoo archipelago in southern Finland, of which 10 islands were small (0.5–4 ha) and five were large (63–966 ha) (Fig. 1). An experimental study was conducted on the 10 small islands which consisted of: (i) the removal of all captured rodents from five of the islands (treatment: removal; designated “removal” islands); and (ii) the release of all captured rodents back to the trapping site (approximately) after sampling and marking on the remaining five small islands (designated “control” islands). Each pair of small islands was randomly allocated to removal and control treatments before the study. The observational study was carried out on the five small “control” islands and the five large islands where the rodents were released after the sampling and marking. Tick collections were also carried out on all islands during the same time period and also during the following year in the spring. An overview of the study design is shown in Fig. 2. Island size, study area and tick collection information are available in Additional file 1: Table S1 and Additional file 1: Table S2. Dung surveys were also carried out at the same time as rodent trappings to estimate the abundance of cervids (Additional file 2: Text S1); however, these surveys had no significant association with the study design, and the dung data are addressed only in the additional files (Additional file 1: Table S3; Additional file 1: Table S4) and the dung index is not included in other models (see section Statistical Analyses).

### Rodent trapping

Rodents were trapped in May, June, July and late August/early September in 2019 using Ugglan live traps (Grahnab AB, Gnosjö, Sweden). The number of trapping squares (4 traps per square, with an inter-trap distance of 20 m) used depended on the island size. On the small islands, we used between two and seven trapping squares (*n*_traps_ = 8–28 per island) to cover the whole island area, while on large islands we used four trapping squares (*n*_traps_ = 16 per island) placed 50–100 m apart from each other; see Additional file 1: Table S1 for more details. Traps baited with sunflower seeds and potatoes were set in the late afternoon at pre-marked locations on each island and checked on two consecutive mornings. All trapping was done within approximately 1 week (May–July: 6 days; August/September: 10 days). In total, we captured three rodent species: bank vole (*Clethrionomys glareolus*), field vole (*Microtus agrestis*) and yellow-necked mouse (*Apodemus flavicollis*). We calculated the rodent abundance index per 100 traps as the sum of bank voles and field voles relative to the trapping effort on the islands (the number of traps per island multiplied by the 2 trapping nights) × 100 to control for the differences in trapping effort between islands. Bank voles and field voles were chosen because they were the most common rodent species on the islands and as their presence varied on different islands, with some islands not having any field voles or bank voles. Yellow-necked mice were not included in the analysis due to the low number of individuals trapped across all islands (*n* = 3).

We determined the species of each rodent and recorded the individual’s characteristics and presence of ectoparasites. An ear biopsy (diameter: 2 mm), taken from one ear of each animal for *B. afzelii* screening, was stored in 70% ethanol at - 20 °C until further processing. Animals captured on the control and large islands were marked with a microchip (Trovan Unique™; Microchips Australia, Keysborough, VIC, Australia) at the first capture and released near their trapping location after sampling. The same data and samples were taken from recaptured animals in the following trapping months (sessions). All animals captured on removal islands were euthanized using cervical dislocation after data collection and sampling.

### Tick collection

Ticks were collected during all rodent trapping sessions and in May 2020. In Finland, tick activity is absent during the winter frost and snow periods. In Finland, depending on the region, life stage and biotope, *I. ricinus* is mostly active from April/May to October/November [35–38]. Both *I. ricinus* larvae and nymphs have been found to be abundant in vegetation in May–June and in August–September in southwest and central Finland [35, 38]. Thus, most of the activity period of the larvae and nymphs was covered by the study period. However, this was the first study to observe the activity dynamics of *I. ricinus* on this island system.

Ticks were collected by dragging a 1-m^2^ cotton flannel flag through the vegetation. After every 10 m, the flag was checked for ticks, and all ticks on the flag were recorded, removed, placed in 70% ethanol and stored at − 20 °C until further processing. The dragging distance depended on the size of the island and the session. On the large islands, the dragging distance was 400 m in all sampling sessions and on all islands in May 2019 and 2020. On the small islands in other sessions (July–August/September), the dragging distance was 100 m to avoid the removal of too many ticks, thus potentially affecting tick abundance and tick-borne pathogen circulation (Fig. 2). Details on the tick collections and infection prevalence per session per island are available in Additional file 1: Table S2. All collected ticks were determined to be *I. ricinus* using a light microscope and morphological identification keys [39–41]. We estimated the average DON per 100 m^2^ per site per session as the number of nymphs collected/dragging distance × 100.

### Detection of *B. afzelii* in ticks and rodents

DNA from nymphs (*n* = 1709) was extracted using the ammonium hydroxide (NH_4_OH) (Sigma Aldrich, St. Louis, MO, USA) method [42] with slight modifications. Each nymph was taken from the 70% ethanol in which it was stored, air dried, and placed in a 2-ml safe lock microtube (Sarstedt, Numbrecht, Germany) containing 200 µl of 1.25% NH_4_OH solution. Each sample was heated for 3 min at 100 °C, crushed using the Qiagen TissueLyser (Qiagen, Hilden, Germany) for 2 min and then incubated at 100 °C for 20 min. After quick cooling, the microtubes were left open and incubated at 100 °C for 15 min to evaporate the ammonia (approx. 50% of the starting volume). Negative controls (containing only 1.25% NH_4_OH solution) were included after every five samples. Total DNA was extracted from rodent ear biopsy samples using the protocol of Laird et al. [43] with negative controls included after every five samples. All extracted DNA was stored at − 20 °C until further analysis.

The DNA samples from the ticks and rodents were screened for *B. burgdorferi* s.l. using real-time PCR (Bio-Rad CFX96 Touch Deep Well Real-Time PCR Detection System; Bio-Rad Laboratories, Hercules, CA, USA). Ticks were screened for *B. burgdorferi* s.l. by targeting 23S ribosomal RNA (rRNA) [44] in most cases, but the flagellin gene [45] was targeted for ticks captured from specific islands in May and June 2019 (Additional file 1: Table S2) as these samples were also included in another study (in preparation). All tick and ear biopsy samples positive for *B. burgdorferi* s.l. were screened using *B. afzelii*-specific primers [46]. The list of primers and probes used is available in Additional file 1: Table S5, while detailed protocols for the PCR assays are provided in Additional file 2: Text S2.

Based on the tick data we calculated the percentage NIP as (the number of quantitative PCR-positive nymphs/total number of screened nymphs) × 100, and the density of infected nymph (DIN per 100 m^2^) as DON × NIP per site per session. For rodents, we calculated the abundance index of infected rodents as (the number of infected animals captured per island divided by trapping effort) × 100 (Additional file 1: Table S1).

### Statistical analyses

First, we evaluated whether rodent removal was successful at controlling rodent abundance. We fitted generalized linear mixed models (GLMMs) with a Poisson distribution using the glmer function in the R package lme4 [47] to examine whether rodent abundance depended on sessions and treatment. The model included the number of rodents captured as the response variable; the trapping effort as an offset; and session, treatment and their interaction as explanatory variables. Island identity was included in the model as a random effect to control for the potential correlation among observations from the same island. An equivalent model was used to examine the role of treatment and session on the abundance of infected rodents in the experimental study. We further studied the response of rodent abundance and the abundance of infected rodents to session and the size category (small vs large) of the island in an observational study. The level of tick infestation of rodents in 2019 was examined in relation to the treatment of the island (removal, control, large), trapping session (May, June, July and August/September) and rodent species (bank voles vs field voles) with individual level rodent trapping data. A GLMM approach with negative binomial distribution and trapping island as the random effect was performed using the glmmTMB package in R [48]. An overview of all the tested models is presented in Additional file 1: Table S6.

On the experimental islands, we investigated whether tick-related measures, i.e. NIP_t+1_, DON_t+1_ and DIN_t+1_, in May 2020 were associated with the rodent removal treatment (removal vs control) or rodent abundance index or abundance index of infected rodents in 2019. We used generalized linear models (GLMs; NIP_t+1_) with binomial error distributions or linear models (LMs; DON_t+1_ and DIN_t+1_). With respect to the NIP_t+1_ and DIN_t+1_ models, we used either rodent removal versus control treatment; the rodent abundance index in 2019 (rodent abundance index of each session tested separately); or the abundance index of infected rodents (each session tested separately). The DON_t+1_ model included the rodent removal treatment versus control treatment, or the rodent abundance index in 2019 (the rodent abundance index with each session tested separately). We tested several NIP_t+1_, DON_t+1_ and DIN_t+1_ models using the rodent abundance index in each trapping session separately, as the rodent abundance index estimates at different sessions were correlated and, thus, cannot be included in the same model. To take into account multiple testing for each response variable (rodent abundance index at four sessions using the same data), the threshold significance level was determined based on Bonferroni correction (0.05/4 = 0.0125), i.e.* P*-value ≤ 0.0125 was considered to indicate a significant result. We also examined DON, NIP and DIN in 2019 to ensure that the treatment (removal vs control) did not differ among the islands before treatment (Additional file 2: Text S3; Additional file 1: Table S6).

On observational islands, we used the same approach as with the experimental islands. We used GLMs and the LM model for testing NIP_t+1_ and DIN_t+1_, respectively, by including the size category (small vs large) of the islands, either the rodent abundance index in 2019 (tested each session separately) or the abundance index of infected rodents in 2019 (each session tested separately) as fixed factors. DON_t+1_ models were analysed using LM including the rodent abundance index in 2019 (tested each session separately) as a fixed factor (Additional file 1: Table S6). To take into account the multiple testing for each response variable, the Bonferroni corrected* P*-value was used in the interpretation of the results (threshold *P*-value = 0.0125).

All data analyses and model building were performed in R (4.2.2) [49].

## Results

### Rodents and tick infestation on the experimental and observational islands in 2019

**Fig. 3:**
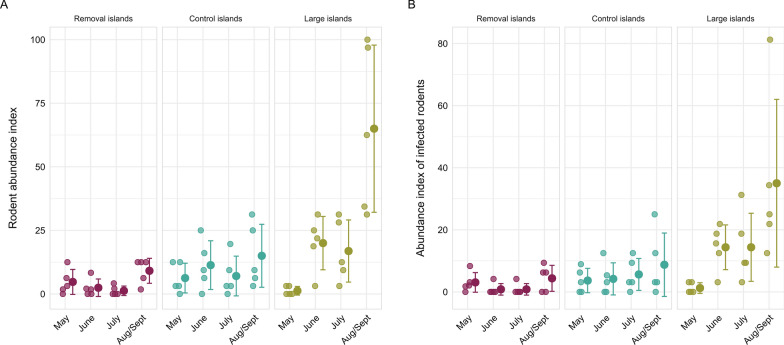
Mean (± standard deviation) rodent abundance index (a) and abundance index of infected rodents (b) on the removal islands, control islands and large islands during the trapping sessions in 2019

Rodent abundance did not differ between the control and removal islands at the beginning of the study (May 2019) (Fig. 3; Additional file 1: Table S7). Rodent abundance increased on the control islands during the breeding season, being significantly higher in the August/September trapping session than in the May trapping session (Fig. 3, Additional file 1: Table S7). Rodent abundance was lower on the removal islands than on the control islands, but the difference was statistically significant only in July 2019 (*P* = 0.04; Additional file 1: Table S7). Additionally, the abundance of infected rodents did not differ between treatments (Additional file 1: Table S7).

Neither rodent abundance nor the abundance of infected rodents differed between the small (control) and large islands at the beginning of the study (May 2019) (Fig. 3; Additional file 1: Table S8). However, the abundances were higher on the large islands than on the small control islands in the other trapping sessions (Fig. 3, *P* < 0.001; Additional file 1: Table S8).

The mean tick infestations on the rodents were 7.6 larvae and 0.56 nymphs per rodent. Of the 281 rodents examined, 246 were infested by ticks (87.5%) and 74 of these (30%) were infested with both larvae and nymphs. Larval tick infestation load on the rodents did not differ between the treatments or the trapping session, but was significantly higher in bank voles than in field voles (Additional file 1: Table S9).

### Ticks and *B. afzelii* in ticks in 2020

**Fig. 4:**
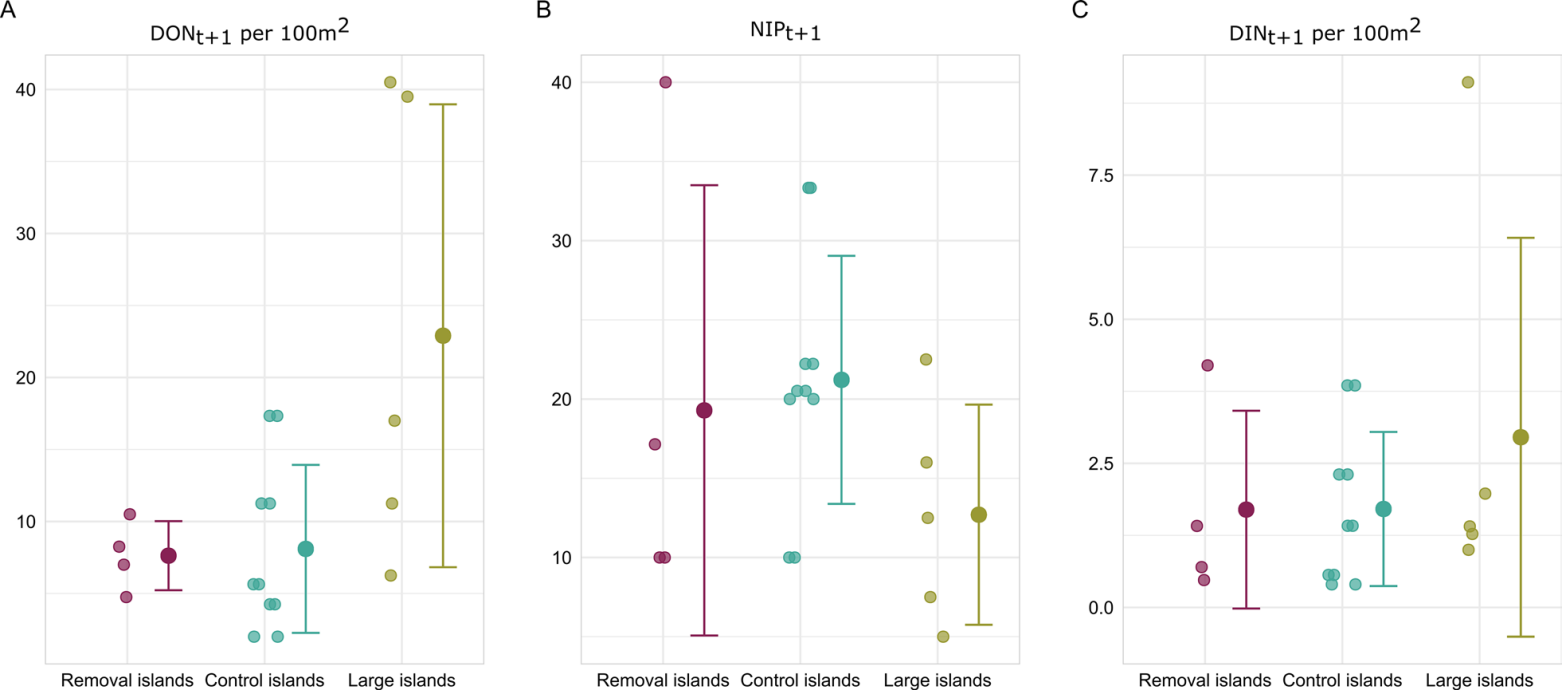
Mean (+/- standard deviation) (a) density of nymphs (DON), (b) nymph infection prevelance (NIP) and (c) density of infected nymphs (DIN) in May 2020 in study islands (removal, control and large). (a) mean DON_t+1_ per 100 m^2^, (b) NIP_t+1_ per 100 m^2^ (c) and DIN_t+1_ per 100 m^2^ in the subsequent year (2020) in removal, control and large islands

In 2020, a total of 550 nymphal ticks were collected. The estimated mean (± standard deviation [SD]) density of nymphs (DON_t+1_) per 100 m^2^ was 6.1 ± 1.79 on the rodent removal islands, 8.09 ± 2.77 on the small control islands and 22.9 ± 7.19 on the large islands (Fig. 4).

In total, 414 of the collected nymphs from May 2020 were tested for *B. afzelii*. In May 2020, the infection prevalence in nymphs (NIP_t+1_) was 15.4% (95% confidence interval [CI]: 3–34%) on the rodent removal islands, 21.2% (95% CI: 11–32%) on the small control islands and 12.7% (95% CI: 4–21%) on the large islands (Fig. 4).

The estimated mean (± SD) density of infected nymphs (DIN_t+1_ = NIP_t+1_*DON_t+1_) per 100 m^2^ in May 2020 was 1.36 ± 0.75 on the rodent removal islands, 1.70 ± 0.63 on the small control islands and 2.95 ± 1.55 on the large islands (Fig. 4).

In 2019, DON, NIP and DIN did not differ between the rodent removal and control islands in any of the sessions, i.e. in May, June, July, and August/September (Additional file 1: Table S10).

### Experimental study: effects of rodents on DON_t+1_, NIP_t+1_ or DIN_t+1_

On the experimental study islands (control and removal islands), there was no effect of rodent removal treatment in the first study year (2019) on DON_t+1,_ NIP_t+1_ and DIN_t+1_ (May 2020) (*P* > 0.5; Additional file 1: Table S11). Within the experimental islands, neither DON_t+1_ (*P* > 0.5; Additional file 1: Table S12) nor NIP_t+1_ (*P* > 0.5; Additional file 1: Table S13) nor DIN_t+1_ (*P* > 0.5; Additional file 1: Table S14) was associated with the rodent abundance index in any of the trapping sessions in 2019. The abundance index of infected rodents in any of the trapping sessions in 2019 were associated with NIP_t+1_ on the experimental study islands (Additional file 1: Table S13).

### Observational study: effects of rodents and island size on, DON_t+1_, NIP_t+1_ or DIN_t+1_

**Fig. 5:**
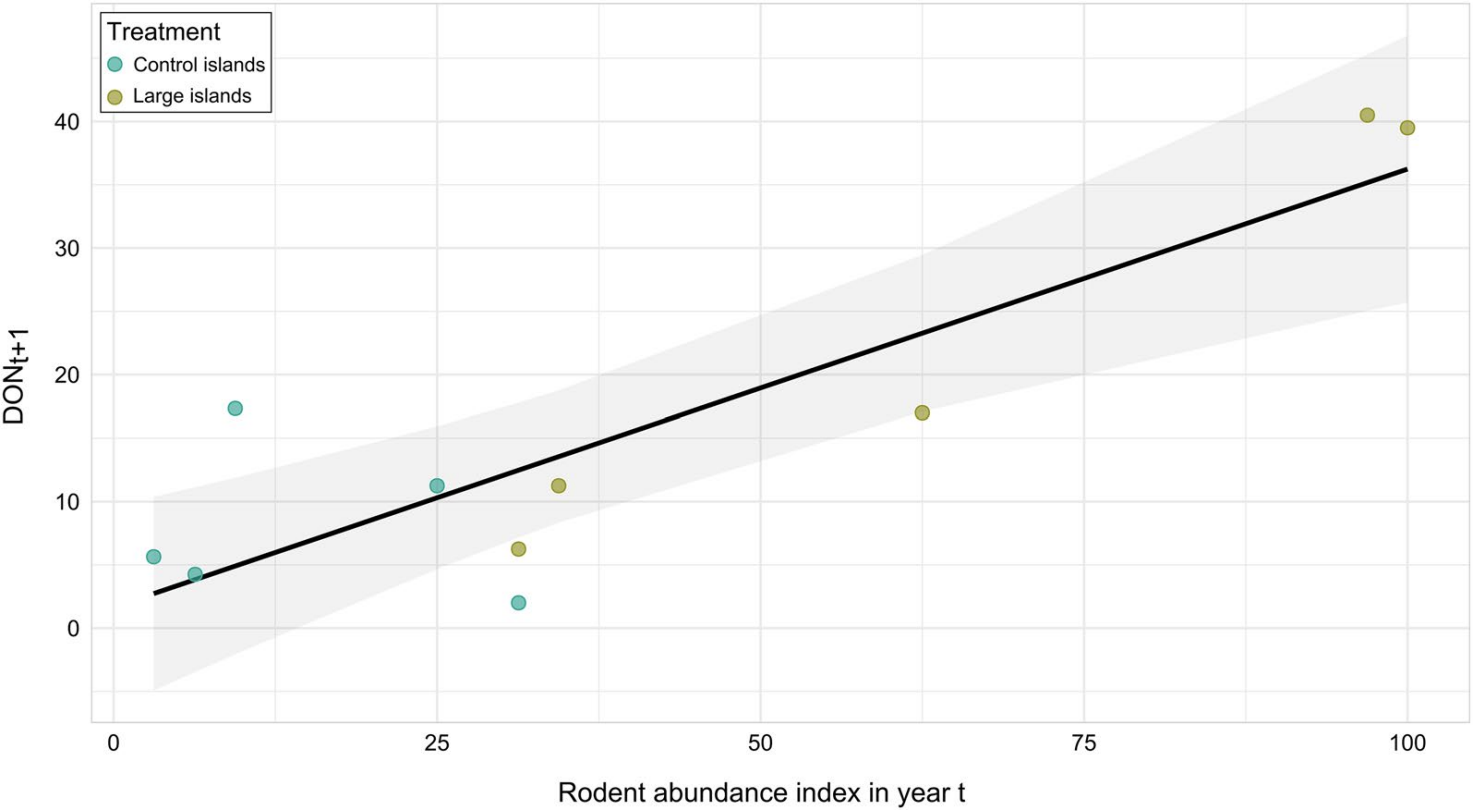
Predicted (line with 95% confidence interval in shading) and observed (points, with different colours indicating the small and large islands, respectively) density of nymphs (DON_t+1_) per 100 m^2^ in May 2020 in relation to the rodent abundance index in August/September 2019 in the observational study. The figure show the relationship between the rodent abundance index in year t (2019) and DON_t+1_ in year t+1 (2020)

On the observational islands (small control and large islands), DON_t+1_ showed a positive association with the rodent abundance index in September (Fig. 5; Additional file 1: Table S12), whereas no association with the rodent abundance index was observed in the other study sessions (Additional file 1: Table S12).

**Fig. 6:**
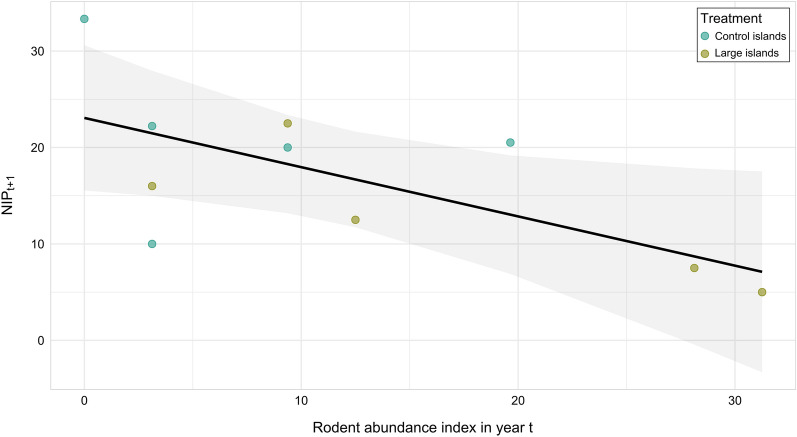
Predicted (line with 95% confidence interval) and observed (points, with different colours indicating the small and large islands, respectively) nymph infection prevalence (NIP_t+1_) in May 2020 in relation to the rodent abundance index in July 2019 in the observational study. The figure show the relationship between the rodent abundance index in year t (2019) and DON_t+1_ in year t+1 (2020)

Moreover, NIP_t+1_ was negatively associated with the rodent abundance index and the abundance index of infected rodents in July 2019 (*P* = 0.011; Fig. 6; Additional file 1: Table S15), but no significant associations were observed during the other trapping sessions. However, DIN_t+1_ was not associated with the rodent abundance index in any trapping session in 2019 (*P*  > 0.5; Additional file 1: Table S16).

Neither DON_t+1_ nor DIN_t+1_ was associated with the island type (Table 1). NIP_t+1_ was associated with island type, with a lower NIP_t+1_ value on large islands (*P* = 0.046; Table 1).

**Table 1:** The estimated effect of island size on NIP_t+1_, DON_t+1_, and DIN_t+1_ in relation to island type (small control islands vs large islands)

Response variable^a^	Explanatory variable^b^	Estimate (standard error)	Z-value	*P*-value
NIP_t+1_	Intercept	− 1.33 (0.22)	− 6.02	< 0.001
	Large island	− 0.63 (0.31)	− 1.99	0.046*
DIN_t+1_	Intercept	1.71 (1.18)	1.44	0.19
	Large island	1.25 (1.67)	0.75	0.48
DON_t+1_	Intercept	8.10 (5.45)	1.49	0.18
	Large island	14.8 (7.70)	1.92	0.09

*Statistically significant

^a^NIP_t+1_ (nymph infection prevalence in following year [2020]) was estimated using generalized linear models (GLMs). DON_t+1_ (density of nymphs in following year [2020]) and DIN_t+1_ (density of infected nymphs the following year [2020]) were estimated using linear models (LM)

^b^Small control islands are included in the intercepts

## Discussion

In this study, we examined the association between the abundance of the reservoir rodent host and the abundance of nymphal *I. ricinus* ticks and a rodent-associated tick-borne pathogen, *B. afzelii*, in ticks. We observed that rodent removal in the experimental study did not translate into changes in the abundance of ticks, tick infection prevalence or the abundance of infected ticks in the following year. Neither NIP_t+1_ nor DIN_t+1_ was associated with the abundance index of rodents or infected rodents on the small experimental islands. In the observational component of this study, we aimed to test whether NIP_t+1_, DON_t+1,_ or DIN_t+1_ was associated with rodent density and island type. Contrary to our hypothesis, we found a negative association between NIP_t+1_ and the abundance index of rodents and infected rodents. However, we found a positive association between rodent abundance index and DON_t+1_.

### NIP_t+1_ in relation to preceding rodent abundances

Our experimental study revealed no association between rodent abundance at t (2019) and NIP_t+1_ (2020). This finding contrasts with the findings of an earlier experimental study by Krawczyk et al. [29] which showed that high rodent abundance was associated with higher infection prevalence of rodent-associated tick-borne pathogens (TBPs) in nymphs. A possible explanation for these differing results is the difference in rodent abundance between the two study systems. Krawczyk and coworkers operated in small (0.25 ha) enclosures, where the highest rodent density was > 40 individuals in an enclosure per year in the removal treatment [29]. Our study was carried out on natural islands that varied in size and had overall lower rodent abundance, with the highest number of captured rodents being up to 15 individuals on a 2-ha removal island. In addition, our tick density was relatively low (on average, < 10 nymphs per 100 m^2^ on removal islands). The low rodent density together with low tick density might decrease the likelihood of larval and nymphal ticks feeding on the same reservoir rodent host individuals, or rodents in general, potentially leading to a lack of association between rodent abundance and NIP_t+1_. However, approximately one third of the rodent individuals were infested with both larvae and nymph(s), which does not provide support to the assumption that low host density and low tick densities decrease aggregation on the host individuals. Nevertheless, our results suggest that even a very low rodent density is sufficient to support the circulation of *B. afzelii*. Alternatively, *B. afzelii* might have been supported by other reservoir species, such as shrews or squirrels [15, 50–54], although the abundance of these species was not estimated reliably in this study. Irrespective of the mechanism supporting pathogen persistence, our results suggest that the removal of rodents even on small islands may not limit the transmission cycle of *B. afzelii* as: (i) we were not able to detect any effect on NIP_t+1_ and (ii) *B. afzelii* was detected on all islands with ticks in May 2020 and rodents 2019. Moreover, although the removal of rodents is unlikely to be a realistic management action for controlling the health hazard caused by *B. afzelii*, it may cause deleterious effects on ecosystems though trophic cascades, such as those affecting predators that prey on rodents.

Interestingly, on the observational study islands, we found a negative association between rodent abundance in the summer and NIP in the following spring. This result contrasts with those from previous studies that showed a positive association between high rodent abundance and nymphal infection prevalence [25–29]. There are several possible reasons for our result. First, it may be that high rodent density could lead to a decrease in tick infection burden on rodents [55, 56], thereby possibly resulting in a decrease infection prevalence in rodents and, ultimately, in decreased NIP_t+1_ in the following year. However, this would be likely only in very low tick densities and very high rodent densities, which were not observed in our study. Although the mean tick infestation load on rodents was considerably lower in our study system than that reported in another northern ecosystem in Norway [57], the rodent density was also low and thus unlikely to decrease tick aggregation on rodents. A second possible explanation is that high rodent abundance in the summer/early autumn likely results from an influx of young, uninfected and/or maternal antibody-protected [58] rodents in the population. Consequently, the high proportion of uninfected rodents potentially limits the transmission of pathogens from rodents to larval ticks. However, the proportion of infected rodents was high throughout our study period. A third potential explanation is that the success of host-tick transmission by *B. afzelii* is limited, as the bank vole, which is the most common rodent in the study system, becomes immune against tick infestation [59, 60].

Lastly, the type of the island in our study was shown to have a significant association with NIP_t+1_, with large islands having lower NIP_t+1_. A previous study conducted in the USA showed that NIP is negatively associated with increasing forest fragment size, indicating the importance of a diverse host community, which can play an important role in tick control and transmission of tick-borne pathogens [61]. As simultaneously large islands had higher rodent abundance, it might be that the negative association between NIP_t+1_ and rodents is caused by some other factor associated with the size of the island. For example, DON was higher on large islands, suggesting that the abundance of hosts that support tick breeding, such as deer, is higher and/or environmental conditions are more suitable for an abundant tick population. Hence, on the larger islands with high tick abundance, a higher proportion of larvae might feed on deer that do not support pathogen transmission, resulting in a decrease in pathogens according to the dilution effect [62–65]. However, we monitored the abundance of dung piles of cervids and found no association either with island type or with DON. Nevertheless, our study highlights the complexity of this disease system and emphasizes the need for further studies to quantify the interplay between rodents, ticks and pathogens, especially in northern Europe.

### Associations of rodent abundance with DON_t+1_ and DIN_t+1_

A positive association between the rodent abundance index and DON_t+1_ was found only in the observational study. Our results are in accordance with those of a previous study that showed a positive association between high rodent abundance and the density of nymphs [27, 35, 66]. This positive relationship might be due to the role that rodents play in the tick life-cycle, acting as a main host for larval feeding and providing good engorgement success for larvae [67], thereby driving nymphal abundance in the following year. An alternative explanation for this positive association is that although cervids were observed on all of our study islands, they may be more persistent on large islands. This persistence could contribute to higher tick density on larger islands, despite the lack of association between DON_t+1_ or the size category of the island, and the abundance of cervid dung piles.

The DIN is a key measure of entomological risk associated with *I. ricinus* because nymphs are the major vectors of human infection. Nymphs are more abundant in nature and more difficult to detect when attached to the host body than adults, while simultaneously carrying several TBPs at relatively high prevalence [11, 13]. Therefore, DIN provides an indication of the risk of human infection with TBPs transmitted by *I. ricinus* nymphs. We expect that the factors influencing DON and NIP can also influence DIN_t+1_, which is a product of DON and NIP [27]. In our observational study on the islands, we found no association between DIN and rodent abundance. This could be because the rodent abundance index has a positive effect on DON but a negative effect on NIP; together, these two opposing effects resulted in a non-significant difference in DIN. Thus, our findings highlight the need for further studies on the fluctuations in the abundance of other vertebrate hosts affecting the density of nymphs and infected nymphs.

## Conclusions

The results of our field experiment study demonstrated that the expected positive associations between rodents and rodent-associated TBPs are not always found. We demonstrated that a reduction in rodent population density may not lead to a corresponding decrease in tick infection prevalence, suggesting that only a few rodents or alternative rodent reservoirs may maintain *B. afzelii* in nature. Consequently, artificial rodent removal may not be sufficient for reducing zoonotic hazards in natural systems. However, our experimental rodent removal and observational rodent trappings were carried out for only 1 year, whereas longer-term studies on islands with larger variations in tick and rodent abundances could provide a better understanding of their relationships in a natural ecosystem. In addition, detailed information on the biodiversity of vertebrate hosts for ticks and pathogens in the ecosystem is required to determine the role of cyclic rodent populations in driving tick and tick-borne pathogens.

### Supplementary Information

**Additional file 1: Table S1. **The population-level data of rodent abundance index and abundance index of infected rodents, including information about trapping sessions and the number of traps. **Table S2. **Population-level data on ticks including information about the number of collected nymphs and infected nymphs during 2019 and 2020. **Table S3.** The estimated effects of the treatments (removal, control and large islands) on the mean density of cervid dung in 2019. **Table S4.** The estimated effects of the treatments (removal, control and large islands) and the average density of cervid dung in 2019 on the density of nymphs on the island (DON_t+1_) in 2020. **Table S5.** Detailed information on primer and probes used for screening of *B. afzelii. ***Table S6. **List of all tested models. **Table S7. **The estimated effects of rodent removal treatment and session (May, June, July and August/September) and their interactions on the rodent abundance and the abundance of infected rodents on the experimental study.** Table S8. **The estimated effects of island size category (small control vs large) and session (May, June, July and August/September) and their interactions on the rodent abundance and the abundance of infected rodents in the observational study. **Table S9.** The estimated effects of the trapping sessions (May, June, July, August/Septe,ber), treatment (removal, control and large islands), and the rodent species (bank vole vs field vole) the larval tick infestation load in 2019.**Table S10.** The estimated effects of the study session (May, June, July, August/September) and rodent removal treatment on the density of nymphs (DON), the density of infected nymphs (DIN) and NIP in 2019 on experimental islands.** Table S11. **The estimated effects of rodent removal treatment on DON_t+1_, NIP_t+1_ and DIN_t+1_ (May 2020) on the experimental islands. **Table S12. **The estimated effects of the rodent abundance index in different trapping sessions (May, June, July and August/September) on DON_t+1_ (May 2020) (**a**) on the experimental and (**b**) on the observational study islands.** Table S13. **The estimated effects of the rodent abundance index and the abundance index of infected rodents in different trapping sessions (May, June, July and August/September) on NIP_t+1_ (2020) on the experimental islands. **Table S14.** The estimated effects of the rodent abundance index and the abundance index of infected rodents in different trapping sessions (May, June, July and August/September) on the density of infected nymphs (DIN_t+1_) in 2020 on the experimental islands. **Table S15.** The estimated effects of the rodent abundance index and the abundance index of infected rodents in different trapping sessions (May, June, July and August/September) on NIP_t+1_ (2020) on the observational islands. **Table S16. **The estimated effects of the rodent abundance index and the abundance index of infected rodents in different trapping sessions (May, June, July and August/September) on the density of infected nymphs (DIN_t+1_) in 2020 on the observational islands.

**Additional file 2: Text S1.** Detailed information on the dung survey and the model used to examine how cervids differ in island treatments (removal, control, and large) and how they affect the density of nymphs (DON_t+1_ **Text S2.** Additional laboratory protocol for PCR. **Text S3.** D Model selection for the effect of treatment on the density of nymphs (DON), the density of infected nymphs (DIN) and nymphs infection prevalence (NIP) in 2019 on experimental islands.

## Data Availability

All population-level data are provided in Additional file 1.

## Abbreviations

DIN: Density of infected nymphs
DON: Density of nymphs
NIP: Nymphal infection prevalence
