# Supplementary material for: Effects of rodent abundance on ticks and Borrelia: results from an experimental and observational study in an island system
Source: Parasit Vectors. 2024 Mar 27;17:157. doi: 10.1186/s13071-024-06130-x (PMC10967205; doi:10.1186/s13071-024-06130-x)
Supplement: Supplementary file 2 — Additional file 2: Text S1. Detailed information on the dung survey and the model used to examine how cervids differ in island treatments (removal, control, and large) and how they affect the density of nymphs (DONt+1 Text S2. Additional laboratory protocol for PCR. Text S3. D Model selection for the effect of treatment on the density of nymphs (DON), the density of infected nymphs (DIN) and nymphs infection prevalence (NIP) in 2019 on experimental islands. [file 13071_2024_6130_MOESM2_ESM.docx]

**Effects of rodent abundance on ticks and *Borrelia* – results from an experimental and observational study in an island system**

Nosheen Kiran^1^, Ilze Brila^1,2^, Tapio Mappes^1^, Saana Sipari^1^, Yingying X.G. Wang^1^, Erin Welsh^1^, Eva R. Kallio^1^

1. Department of Biological and Environmental Sciences, University of Jyväskylä, Finland
2. Ecology and Genetics Research Unit, University of Oulu, Oulu, 90014, Finland

**Keywords:** *Ixodes ricinus*, *Borrelia burgdorferi, Borrelia afzelii,* nymphs, infection prevalence, host abundance.

**Supplementary material and method:**

**TEXT S1: Dung survey**

Dung surveys were carried out by walking along the edges of rodent trapping quadrats (20 meters interval between traps and 4 traps, in total 80 meters per quadrat), for 2-6 quadrats per site per session, and observing all dung piles in a one-meter-wide track. The most common species observed based on the dung were white-tail deer (WTD; *Odocoileus virginianus*), roe deer (RD; *Capreolus capreolus*) and moose (*Alces alces*). As distinguishing WTD and RD fecal pellets to species level was difficult and due to the similar role of WTD and RD as reproduction hosts for *I. ricinus*, we summed the data from WTD and RD and used a deer. Deer and moose are expected to have similar roles for ticks and *B. burgdorferi* s. l., and hence they were combined as cervids. Cervid dung pile number/100 meters was counted by summing the deer and moose dung piles observed per session, divided by the total distance of dung surveys, and multiplied by 100 meters for the analyses.

We used a linear model to investigate whether the abundances of cervid dung differ between the island treatments, i.e., small removal islands, small control islands or large islands. There were no significant differences in cervid dung abundance between the treatments (Additional file 1: Table S3). Consequently, cervids were excluded from further analysis. Additionally, we utilized a linear model to whether DON_t+1_ (2020) was associated with cervid dung abundance in 2019 and the treatment of the island (removal, control, large) (Additional file 1: Table S4). The results suggest no association between DON_t+1_ and cervid dung abundance (Additional file 1: Table S4).

**TEXT S2: Additional laboratory protocols**

The primers and probes used in the analyses of *B. burgdorferi* s. l. are reported in additional file 1: Table S5. The PCR master mix consisted of 7.5 µl of the ITaq universal Probes Supermix (Bio-rad, USA), 10 µM of primers (forward and reverse) and probe, 2 µl of Bovine albumin serum (BSA) (5 mg/ml) and 3.5 µl of template DNA, made up to a final volume of 15 µl with sterile molecular grade water. The thermal cycling profile used for qPCR analyses of the pathogen was 5 min denaturation at 95 °C, followed by 50 cycles for *B. burgdorferi* s.l. and 45 cycles for *B*. *afzelii* at 95 °C for 10 s and 60 °C for 1 min.

A positive control (cultured positive control), negative extraction controls, and negative controls (with graded water only) were included in each plate. If any of the negative controls from extraction showed a positive signal in the qPCR, they were rerun together with 5 samples before and after the potentially contaminated negative controls. After this repeat, no negative controls were detected as contaminated.

**TEXT S3: Effect of treatment on DON, NIP, and DIN from 2019**

We examined the density of nymphs (DON), *B. afzelii* infection prevalence in nymphs (NIP), and the density of infected nymphs (DIN = DON*NIP) in 2019. NIP was examined using a generalized linear model (GLMM) with binomial response variable (number of infected and uninfected nymphs) using session (May, June, July, and August/September), treatment (removal vs control) as fixed factors, and island ID as a random factor. DON and DIN were examined using a linear mixed model (LMM) (lme function in nlme package (Pinheiro et al., 2007)) with the same fixed and random factors as in the NIP model (Additional file 1: Table S10).
